# Insights from the Genome Annotation of *Elizabethkingia anophelis* from the Malaria Vector *Anopheles gambiae*

**DOI:** 10.1371/journal.pone.0097715

**Published:** 2014-05-19

**Authors:** Phanidhar Kukutla, Bo G. Lindberg, Dong Pei, Melanie Rayl, Wanqin Yu, Matthew Steritz, Ingrid Faye, Jiannong Xu

**Affiliations:** 1 Biology Department, New Mexico State University, Las Cruces, New Mexico, United States of America; 2 Department of Molecular Biosciences, The Wenner-Gren Institute, Stockholm University, Stockholm, Sweden; Virginia Tech, United States of America

## Abstract

*Elizabethkingia anophelis* is a dominant bacterial species in the gut ecosystem of the malaria vector mosquito *Anopheles gambiae*. We recently sequenced the genomes of two strains of *E. anophelis*, R26^T^ and Ag1, isolated from different strains of *A. gambiae*. The two bacterial strains are identical with a few exceptions. Phylogenetically, *Elizabethkingia* is closer to *Chryseobacterium* and *Riemerella* than to *Flavobacterium.* In line with other Bacteroidetes known to utilize various polymers in their ecological niches, the *E. anophelis* genome contains numerous TonB dependent transporters with various substrate specificities. In addition, several genes belonging to the polysaccharide utilization system and the glycoside hydrolase family were identified that could potentially be of benefit for the mosquito carbohydrate metabolism. In agreement with previous reports of broad antibiotic resistance in *E. anophelis*, a large number of genes encoding efflux pumps and β-lactamases are present in the genome. The component genes of resistance-nodulation-division type efflux pumps were found to be syntenic and conserved in different taxa of Bacteroidetes. The bacterium also displays hemolytic activity and encodes several hemolysins that may participate in the digestion of erythrocytes in the mosquito gut. At the same time, the OxyR regulon and antioxidant genes could provide defense against the oxidative stress that is associated with blood digestion. The genome annotation and comparative genomic analysis revealed functional characteristics associated with the symbiotic relationship with the mosquito host.

## Introduction

The mosquito gut accommodates a diverse and dynamic microbiota [1]–[5], which has a profound impact on host metabolism, fecundity [6] and immunity [7], [8]. The gut microbiome is not a random assemblage; the common core taxa belong to Proteobacteria, Bacteroidetes and Actinobacteria [2]. To better understand its structure and function in the mosquito gut ecosystem, it is necessary to characterize abundant taxa. *Elizabethkingia* is a genus in the Flavobacteriaceae family of Bacteroidetes and represents a separate lineage from the *Chryseobacterium-Bergeyella-Riemerella* branch [9]. *Elizabethkingia* spp. has been found to be a predominant resident in the gut of *Anopheles gambiae* [1], [2], *An. stephensi* [4], [10] and *Aedes aegypti* [11]. Lately, the strain R26^T^ of *Elizabethkingia* sp. was isolated from the midgut of *An. gambiae*, Ifakara strain [1]. The comparison of 16S ribosomal RNA (rRNA) gene sequences indicated closest similarity (98.6%) to that of *Elizabethkingia meningoseptica*. Subsequent hybridization experiments and fingerprint analyses together with biochemical tests, however, clearly separated R26^T^ from *E. meningoseptica* and *E. miricola*. Hence, a novel species, *Elizabethkingia anophelis*, was proposed as a third member in the genus *Elizabethkingia* [12]. Similar to *E. meningoseptica*, *E. anophelis* is a non-motile, non-spore-forming Gram negative rod with natural resistance against several antibiotics [12]. Later, the strain Ag1 of *E. anophelis* was isolated from the G3 strain of *An. gambiae.* The genomes of both strains were sequenced and annotated [13]. Here we present the *in silico* annotation of the genome, particularly focusing on functional categories that provide insights regarding the ecological connection between the bacteria and the mosquito host.

## Materials and Methods

### Genome Project History

The strains of R26^T^ and Ag1 of *E. anophelis* were isolated from the midgut of *Anopheles gambiae* maintained in the Faye laboratory in Stockholm University [1], [12] and the Xu laboratory in New Mexico State University, respectively. The genome sequencing, assembly and gene prediction were described in [13]. The draft genomes have been deposited at DDBJ/EMBL/GenBank. The R26^T^ and Ag1 genomes are under the accession ANIW01000000 and AHHG00000000, respectively.

### Functional Categorization

The predicted genes were functionally categorized using SEED subsystems [14] at the RAST server (http://rast.nmpdr.org) [15]. Conserved functional domains in protein sequences were identified using NCBI Conserved Domain Search Service (CD Search) [16]. Genes encoding candidate carbohydrate active enzymes, including enzymes of assembly (glycosyltransferases, GT) and deconstruction (glycoside hydrolases, GH, polysaccharide lyases, PL, carbohydrate esterases, CE) were detected with the CAZymes Analysis Toolkit (CAT) [17] using the Carbohydrate Active Enzyme (CAZy) database [18], [19].

### Genomes used in the Analysis

The *E. anophelis* genome was compared to several available genomes in the Flavobacteriaceae family: *E. meningoseptica* ATCC 13253 (BioProject: PRJDB229), *Chryseobacterium gleum* ATCC 35910 (BioProject: PRJNA30953), *Chryseobacterium* sp. CF314 (BioProject: PRJNA83055) [20], *Flavobacterium johnsoniae* UW101 (BioProject: PRJNA58493) [21], *Flavobacterium branchiophilum* FL-15 (BioProject: PRJNA73421 [22] and *Riemerella anatipestifer* RA-CH-2 (BioProject: PRJNA183917). The bacterial 16S rDNA and *rpoB*, the gene that encodes the β subunit of bacterial RNA polymerase, were used to investigate phylogenetic relationships between the species in the Flavobacteriaceae family. The Multiple protein sequences were aligned using ClustalW and a neighbor joining phylogeny tree was constructed using MEGA 5.10 [23]. The Gamma distribution of substitution rate, Jones-Taylor-Thornton (JTT) model and pairwise deletion of gap/missing data were chosen for the tree construction. Bootstrap was conducted with 1000 replications. Synteny analysis was performed using Mauve Multiple Genome Alignment software [24]. A PCR assay was designed to verify an Ag1-present and R26^T^- absent segment. Primer sequences were: F1, CGGATCTTTTAATACCCAGCGT; R1, GGCATTTCCTGTCGTTACACC; F2, AACCTGCTGAACCTACAACGG; R2, GCCAATCTGTAAGTAGCGCC (Fig. S1).

### Antimicrobial and Hemolysis Assays

Antibiotics and Cecropin A from *Hyalophora cecropia* were purchased from Sigma-Aldrich. The radial diffusion assay was performed as earlier described by Hultmark et al [25]. Blood agar plates (horse blood) and bacterial control strains were kindly provided by Ann-Beth Jonsson’s lab group. Hemolytic activity was evaluated following incubation at 37°C for up to 48 h.

## Results and Discussion

### Genome Properties

**Figure 1:**
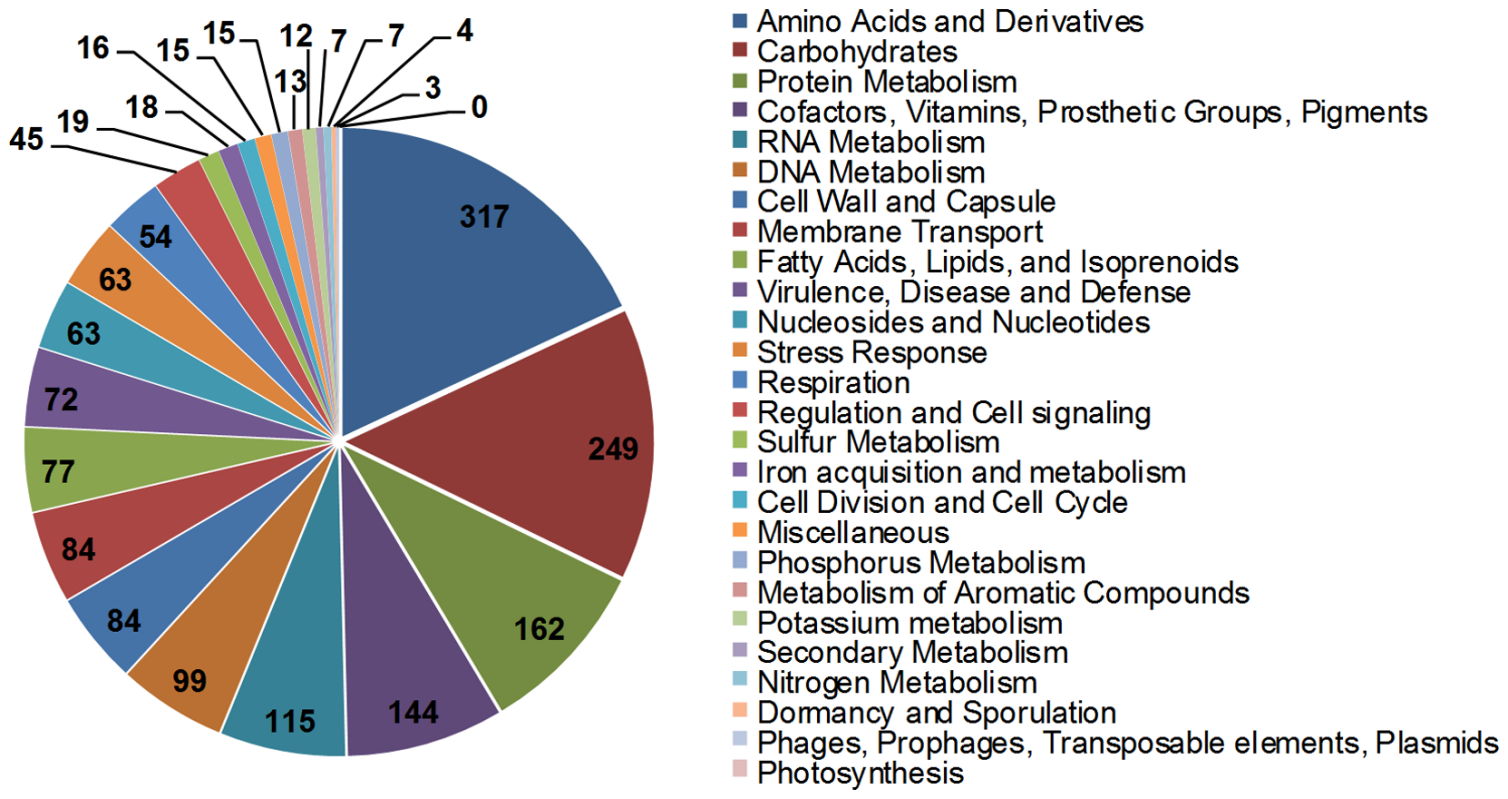
Subsystem category distribution statistics for the genome of *E. Anophelis* as annotated by RAST. The pie chart represents relative abundance of each subsystem category and numbers depict subsystem feature counts.

The draft genome of type strain R26^T^ of *Elizabethkingia anophelis* is 4.03 Mbp in size with an average GC content of 35.4%. The genome was predicted to have 3687 protein coding sequences (CDS) and 44 RNA genes. A single copy of a 16 S rRNA gene is found in the genome. The genome statistics are presented in Table S1. Among the predicted protein coding genes, 2169 were assigned a putative function and 1518 were hypothetical proteins. To characterize the functions of the genome, the draft genome was annotated at RAST using the SEED subsystems [15]. Among the protein coding genes, 1174 were assigned into 308 subsystems (Fig. 1). The gene name, locus tag, protein ID and similarity comparison between the R26^T^ and Ag1 genomes are presented in Table S2. The genomes of Ag1 and R26^T^ are almost identical with a few exceptions. A small number of predicted ORFs (185 in R26^T^ and 146 in Ag1) were not found in the other strain, likely because both genomes were incomplete. Strain specific genomic regions were, however, identified. Two R26^T^ contigs, 104 (ANIW01000041; 28,343 bp) and 107 (ANIW01000035; 5,773 bp), are not found in the Ag1 genome. The R26^T^ contig 104 contains six putative genes associated with conjugal elements, indicating the presence of a conjugative plasmid or transposon. In addition, a segment of 32,350 bp in the Ag1 contig 47 (AHHG01000028) is not present in the counterpart contig 16 (ANIW01000059) in the R26^T^ genome. The presence of putative phage genes in the Ag1-segment indicates presence, or remnants, of a lysogenic phage. The insertion/deletion between Ag1 contig 47 and R26^T^ contig 16 was verified by PCR and subsequent sequencing of the amplicons (Fig. S1). Further investigation is necessary to confirm the identity and nature of those strain-specific elements.

### Phylogenetic Relationship to Related Genomes

Upon comparison to available genomes in the family Flavobacteriaceae, *E. anophelis* is closer to *E. meningoseptica, Riemerella anatipestifer* RH-CH-2, *Chryseobacterium gleum* ATCC 35910 and *Chryseobacterium* sp. CF314 [20] and is divergent to taxa in the genus *Flavobacterium*. Table 1 shows the similarity of comparable CDS between *E. anophelis* R26^T^ and six other genomes. The pattern was supported by the phylogenetic relationship inferred from the 16S rRNA (Fig. S2A) and the peptide sequences of RNA polymerase beta-subunit, rpoB (Fig. S2B). *E. anophelis* and *E. meningoseptica* are grouped as a clade sister to the genera *Chryseobacterium* and *Riemerella,* while the taxa of the genus *Flavobacterium* are clustered in a separate clade.

**Table 1:** The similarity of comparable CDS between R26^T^ and related genomes in the family Flavobacteriaceae.

Species	Identity			
	Comparable CDS	80–100%	50–79.9%	20–49.9%
*Elizabethkingia meningoseptica* ATCC 13253	3791	2481 (65.4)	940 (24.8)	370 (9.8)
*Chryseobacterium gleum* ATCC 35910	2921	578 (19.8)	1537 (52.6)	806 (27.6)
*Chryseobacterium* sp. CF314	2773	521 (18.8)	1382 (49.8)	870 (31.4)
*Flavobacteriaceae bacterium* 3519–10	2299	321 (14.0)	1077 (46.8)	901 (39.2)
*Riemerella anatipestifer* RA-CH-2	2021	306 (15.1)	883 (28.6)	832 (56.3)
*Flavobacterium johnsoniae* UW101	2569	34 (1.3)	1001 (39.0)	1534 (59.7)

Numbers within parenthesis reflect the percentage of total comparable CDS.

### TonB Dependent Transporters

The bacterial TonB-dependent transporters (TBDTs) are specialized elaborate machinery for active uptake of rare but essential nutrients and other substrates, such as iron complexes, vitamin B12, nickel, carbohydrates and colicin [26]–[30]. The total number of TBDTs is highly variable among bacterial genomes. Recently, Mirus *et al.* (2009) found putative TBDTs in 347 out of 686 sequenced bacterial genomes [31]. Among the investigated taxa of Bacteroidetes, all were found to endow over 50 *TBDT* genes. This is in line with our finding of 59 *TBDT* genes in *E. anophelis*. In the two other bacterial genomes that were isolated from the gut of *An. gambiae* in the Xu lab, *Pseudomonas* sp. Ag1 and *Enterobacter* sp. Ag1 possessed 55 and 20 *TBDT* genes, respectively [32], [33]. Different TBDTs are distinct in substrate specificity [31], [34]. The protein conserved domain analysis revealed various domains associated with different substrates. The conserved domains of 14 representative TBDTs are presented in Fig. S3. The architecture of mosaic conserved domains implies sophisticated interactions of TBDTs and biopolymers. The various TBDTs endow the bacterium with an uptake system for a variety of biopolymers, which have been demonstrated in many Gram negative bacteria, including Bacteroidetes in different metagenomic settings [35]–[37]. Further investigations are necessary to characterize the substrates of the TBDTs of *E. anophelis* and their contributions in the gut microbial community.

**Figure 2:**
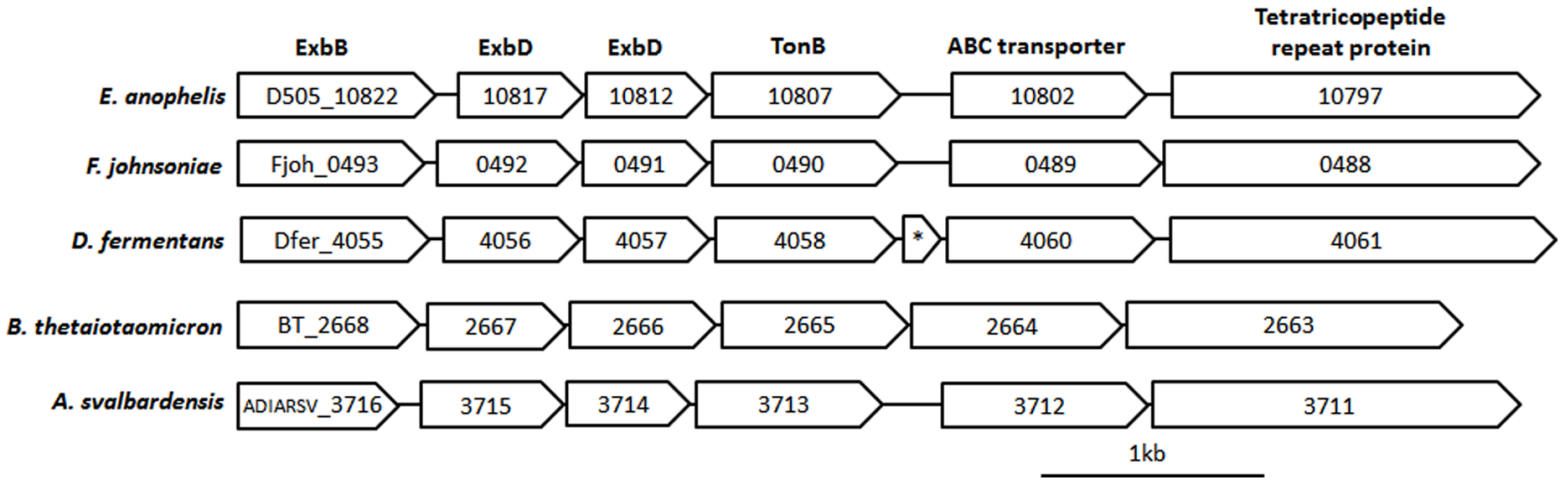
Graphic view of a syntenic gene cluster that is conserved in five taxa of Bacteroidetes. The genes encoding the components of TonB dependent transporters are ExbB, ExbD and TonB. Locus ID of each gene is given in the boxes, with taxon prefix (e.g. D505_ for *E.a.*) in the ExbB box for each species. The box with * in *D. fermentans* represents a predicted gene encoding a hypothetic protein. The scale bar represents 1 kb in length. Phylogenetically, *E. anophelis* and *F. johnsoniae* belong to the class Flavobacteria, *Dyadobacter fermentans* belongs to the class Cytophagia, *Arcticibacter svalbardensis* is in the class Sphingobacteriia, and *Bacteroides thetaiotaomicron* is located in the class Bacteroidia.

To energize the transport process, TBDTs interact with the TonB complex, a cytoplasmic transmembrane assembly of the proteins ExbB and ExbD, which couples with the TonB in periplasm. In the genome of *E. anophelis,* two *ExbB*, two *ExbD* genes and four *TonB* genes were found. A gene cluster was identified in the genome, where *ExbB, ExbD* and *TonB* as well as genes encoding an ABC transporter and a tetratricopeptide repeat containing protein are co-localized. The synteny of the gene cluster is conserved in taxa from all of the four existing classes of Bacteroidetes (Fig. 2). Such conservation indicates that it originated from a common ancestor and remained as an inheritable unit due to functional relationships among these syntenic genes.

### Polysaccharide Utilization Loci

**Figure 3:**
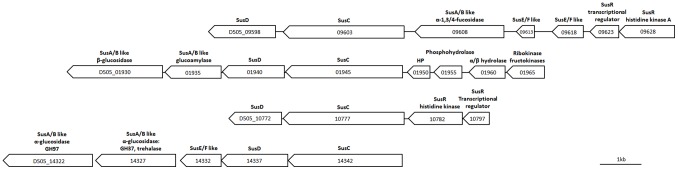
Graphic view of four Sus-like loci in the genome of *E. anophelis* R26^T^. Locus ID of each gene is given in the boxes. HP: hypothetic protein. The scale bar represents 1 kb in length.

Carbohydrates serve as a major carbon and energy source for bacteria. In Bacteroidetes, utilization of complex carbohydrates (glycans) involves polysaccharide sensing, degrading and import machinery. Such activities are mainly controlled by genes in various polysaccharide utilization loci (PULs) [29], [38]–[41]. The starch-utilization system (Sus) represents a typical example of glycan acquisition [29], [42]. The Sus like PUL consists of a cluster of genes encoding SusD (glycan-binding protein), SusC (Ton-B dependent transporter), SusE/SusF (carbohydrate-binding proteins without enzyme activity), SusA, SusB, SusG (enzymes for polysaccharide deconstruction) and SusR (an inner membrane-associated sensor-regulator system for transcriptional activation of Sus genes) [29], [42]. The genome of *E. anophelis* contains 28 pairs of SusD and SusC homologs. Fig. 3 shows four loci where putative Sus-like genes are located. Table S3 lists predicted CAZy proteins in the *E. anophelis* genome, including glycoside hydrolases (GH), such as α-glucosidase, β-glucosidases, α-galactosidase, β-galactosidase, α-mannosidase, α-amylase, β-mannosidase, β-glucanase, cellulase, maltodextrin glucosidase, xylosidase, and α-dextrin endo-1,6-α-glucosidase, which indicate a broad capability of degrading polysaccharides. Recently, Kolton *et al.* (2013) demonstrated that taxa in the genus *Flavobacterium* were separated into terrestrial and aquatic clades according to geographical distribution. The terrestrial taxa have greater capacity to degrade polysaccharides [43]. Interestingly, some CAZy enzymes of R26^T^ have orthologs in *F. johnsoniae*, a terrestrial taxon, but not in the aquatic taxon *F. branchiophilum*. It is thus plausible that *Elizabethkingia* species have a similar ecological niche as the terrestrial taxa of flavobacteria.

Sucrose and fructose are the most common sugars that mosquitoes ingest from floral nectar [44], [45]. Digestion of the sugar is carried out mainly by α-glucosidases that catalyze the hydrolysis of 1,4-α-glucosidic bonds to release α-glucose. The mosquito α-glucosidases have been characterized [46], [47]. In addition, mosquitoes can ingest plant tissue and cellulose particles from plants, especially in arid habitats where floral nectar is scarcely available [48]–[50]. Certain polysaccharides from plants (e.g. hemicelluloses, pectins, and starch) may contain several different monosaccharides and a variety of glycosidic linkages. Utilization of the polysaccharides with such a wide structural variety and fluctuating abundance requires sophisticated mechanisms to recognize and degrade them [41]. The predominance of *E. anophelis* in the sugar fed gut [2] and the possession of numerous Sus-like loci and GHs suggest that the bacterium may be capable of utilizing plant cellulose in the diet. Interestingly, no SusC and SusD were found in the genomes of *Enterobacter* and *Pseudomonas* strains that were isolated from the mosquito gut [32], [33]. The large capacity of polymer transport and utilization implies a potential ecological association between the mosquito and the gut microbiota in which certain microbial residents may support effective utilization of various types of polysaccharides that mosquitoes take in from nectar-rich or nectar-poor plants.

### Antibiotic Resistance

**Figure 4:**
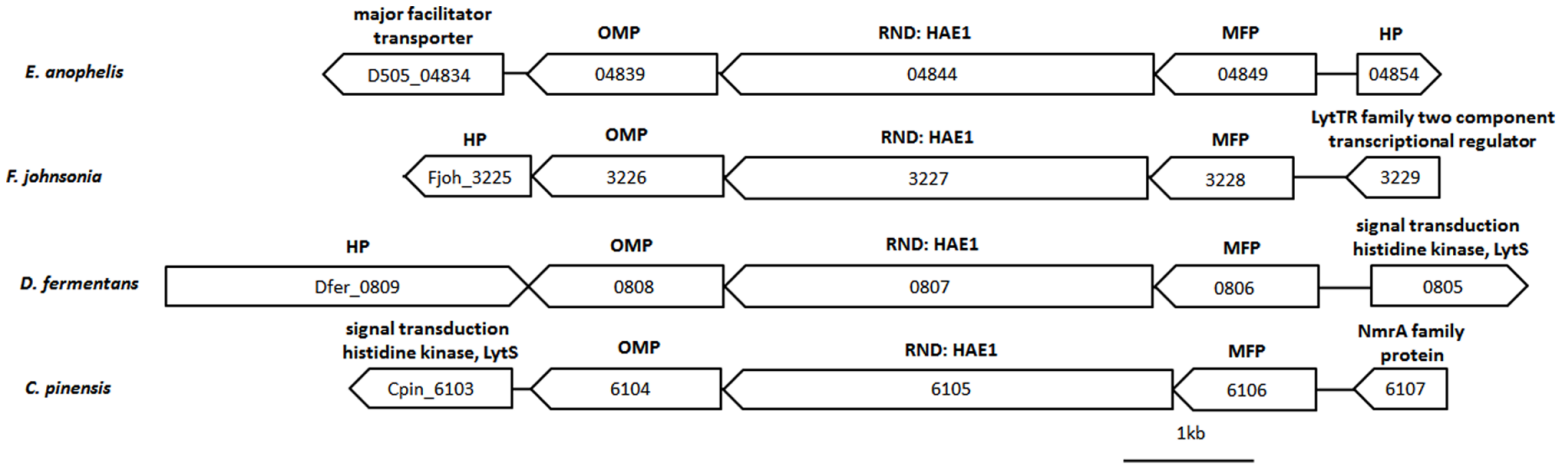
Graphic view of a locus where three component genes of an RND efflux pump are located syntenically in four taxa of Bacteroidetes. Gene name was given on the top of each box. Locus ID of each gene is given in the boxes. OMP, outer membrane protein; RND: HAE1, Hydrophobe/amphiphile efflux-1; MFP, membrane fusion protein; HP: hypothetic protein. The scale bar represents 1 kb in length.

*E. anophelis* is known for its intrinsic resistance to many antibiotics [12]. The resistance mechanisms employed by bacteria are manifold, such as the enzymatic degradation of the drug, the alteration of the target drug site and the direct extrusion of the drug from the cells through efflux pumps. The *E. anophelis* genome appears to contain a large number of resistance genes (Table 2, S4). This includes a large set of multidrug efflux pumps, predominantly members of the resistance-nodulation-division (RND) and major facilitator multidrug pumps (MFS) families. The RND transporters can mediate extrusion of a broad range of substrates, including heavy metals (heavy metal efflux, HME), multidrug hydrophobe/amphiphile efflux-1 (HAE1) and toxic chemical compounds to maintain homeostasis [51], [52]. The RND efflux pump is a tripartite assembly composed of an inner RND transport protein, a membrane fusion protein (MFP) and an outer membrane protein [53], [54]. There are 13 sets of genes encoding the components of the RND pumps in the genome of *E. anophelis*. In most cases, three genes were found adjacent to each other, likely in a single operon. The synteny is conserved in the flavobacteria compared in this study as well as in other taxa of Bacterioidetes (Fig. 4, Table S4). The role of RND-efflux pumps in intrinsic antibiotic resistance has been demonstrated in *Chryseobacterium* and *F. johnsoniae* [55], [56], as well as in *Bacteroides fragilis* [57]. The genome also encompasses genes encoding 44 MFS and two multidrug and toxic compound extrusion (MATE) transporters (Table S4). Generally, the MFS proteins mediate transport of a wide spectrum of substrates, including ions, carbohydrates, lipids, amino acids and peptides, nucleosides, and other molecules [58], [59].

**Table 2:** Summary of the functional subcategories of resistance genes.

	Number of Genes	Average identity (%)			
Functional classification		Ag1	*E. m.*	*C. g.*	*F. b.*
RND efflux pump	37	100	83.5	66.5	27.7
MFS transport	44	100	73.7	55.5	25.1
Resistance to β-lactams	28	100	69.0	52.4	24.2
Resistance to fluoroquinolones	4	100	96.7	82.9	63.1
Drug resistance: other	19	100	76.8	52.1	17.8
Heavy metal detoxification	11	99.3	77.4	68.3	39.6

Average identity reflects the mean identity of genes within the subcategory compared to R26^T^. *E. m*., *Elizabethkingia meningoseptica*; *C. g.*, *Chryseobacterium gleum*; *F. b., Flavobacterium branchiophilum*.

**Figure 5:**
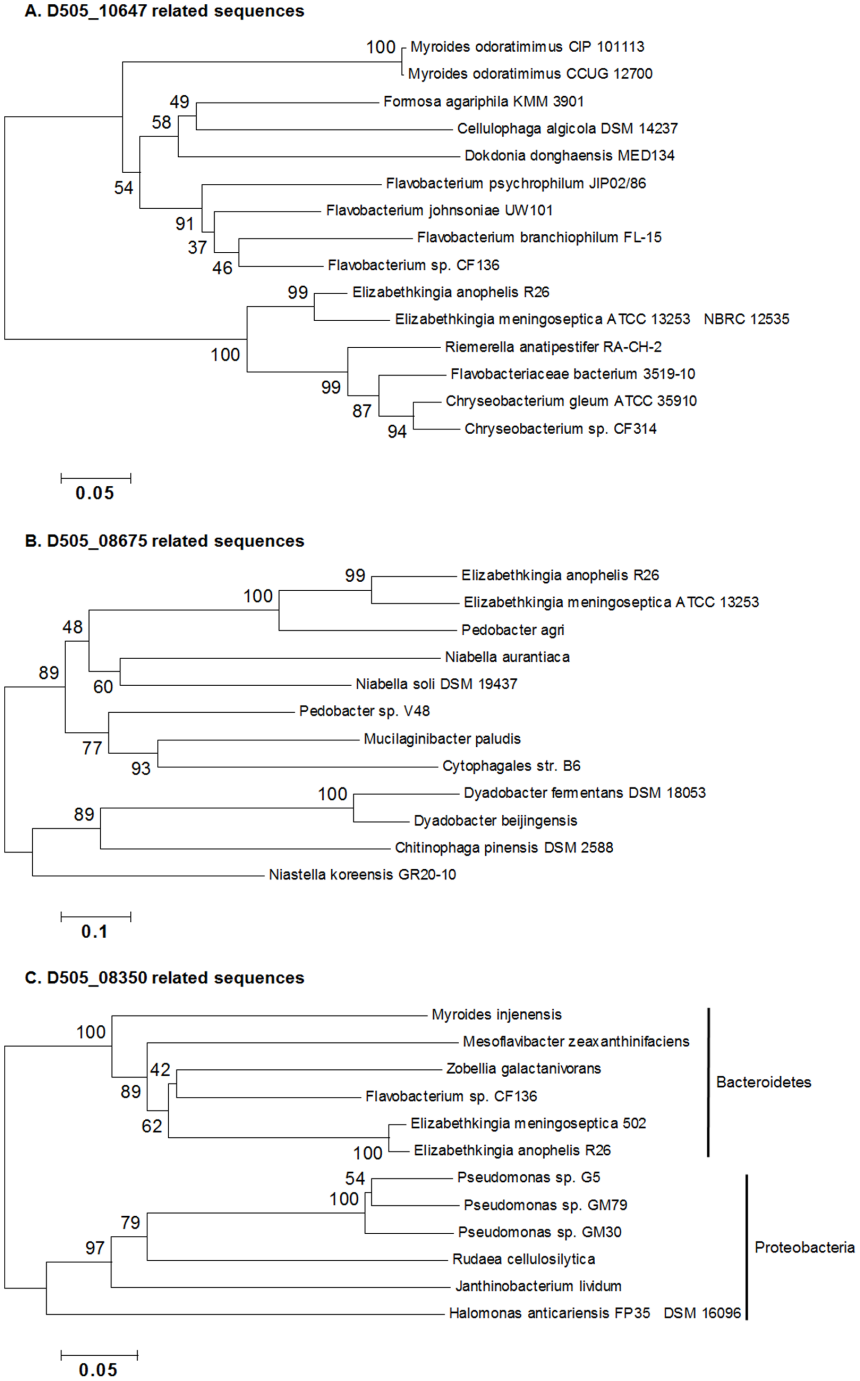
Phylogenetic relationship of homologues of three selected lactam degrading enzymes in *E. anophelis* and other taxa. (A) D505_10647; (B) D505_08675; (C) D505_08350. Numbers above clades are bootstrap values (1,000 replicates). The trees were constructed by Neighbor Joining criterion implemented in MEGA 5.1. The GenBank accession numbers of the sequences were listed in Table S5.

The synthesis of β-lactamases is the most commonly employed strategy among Gram-negative bacteria to combat β-lactam antibiotics [60]. A large set of genes conferring resistance to β-lactams were annotated in the R26^T^ genome, including 19 β-lactamases, four metallo-β-lactamases (MBLs) and four penicillin-binding proteins (Table S4). An overall lower degree of conservation between the compared species and R26^T^ was observed in this subcategory. Phylogenetic analysis of selected lactam degrading enzymes suggests that in addition to vertical inheritance, certain β-lactamase genes may be acquired by lateral gene transfer. As an example of the former, the β-lactamase D505_10647 appears conserved and vertically transmitted in Flavobacteriaceae (Fig. 5A, Table S5). In contrast, D505_08675 is not found in other members of Flavobacteriaceae except *E. meningoseptica.* Orthologs are instead present in the taxa belonging to the class Sphingobacteria of Bacteroidetes (Fig. 5B). Moreover, the MBL encoding gene D505_08350 lacks orthologs in several closely related species including *E. meningoseptica* ATCC 13253 (Fig. 5C), and appears to share common ancestry with the homologues in some Proteobacteria, particularly of the genus *Pseudomonas*, which indicates a history of lateral transmission. It is noteworthy that *Pseudomonas aeruginosa* produces transferable MBLs that recently have been spread to Enterobacteriaceae, likely in a clinical environment [61]. MBLs are also present in environmental microbiota, suggesting that certain taxa, including *E. anophelis*, potentially could act as reservoirs for lateral gene transfers in nature [62]–[65]. These patterns suggest that the β-lactamase superfamily is adaptive in response to various antimicrobial compounds in different eco-contexts.

*E. meningoseptica* endows two families of wide spectrum MBLs termed GOB [66], [67] and BlaB [68]. Of note, an exceptional genetic diversity of the family members has been reported between clinical isolates from Korea [69]. In line with this, we repeatedly observed a clearly stronger homology for the lactam degrading enzymes between R26^T^ and *E. meningoseptica* strain 502 than the type strain ATCC 13253 (data not shown). This included the putative GOB/BlaB members, of which most are present *E. meningoseptica* 502 but only a few in ATCC 13253 [70]. This led us to compare the genome-wide CDS similarities between these two strains and R26^T^. In comparison to *E. anophelis*, 85.3% and 69.3% of CDS in *E. meningoseptica* 502 and ATCC 13253, respectively, display >80% identity. A similar comparison against CDS of *E. meningoseptica* 502 revealed 85.1% identity to R26^T^ and only 58.7% to ATCC 13253. This unequivocally suggests that *E. meningoseptica* 502 is more related, albeit not identical to *E. anophelis* and raises concerns of potential misinterpretations when classifying strains of the genus. More thorough methods are hence needed in order to accurately determine whether isolates of *Elizabethkingia* sp. belong to either of the described species or represent a novel taxon.

The broad genetic capacity for antibiotic resistance is consistent with the observation that *E. anophelis* R26^T^ has natural antibiotic resistance to ampicillin, chloramphenicol, kanamycin, streptomycin and tetracycline [12]. In addition, ciprofloxacin that previously has proven moderately potent against clinical isolates of *E. meningoseptica* [71]–[73] had limited effects on R26^T^ growth (≥50 µg × ml^−1^), whereas rifampicin [73], [74] displayed cytotoxicity at moderate doses (≥3.125 µg × ml^−1^). A strong resistance against the insect antimicrobial peptide Cecropin A was also seen, as no bacterial clearance was observed at the highest dose (100 µM; Fig. S4).

The interactions among antibiotic-producing and resistant bacteria may be one of the determinants that shape and stabilize the community structure in the mosquito gut. Similar metagenomic contexts have been demonstrated in natural environments [75] and host associated microbiomes [76], [77]. The inhabitation of multidrug resistant *E. anophelis* and possibly other bacteria in the mosquito gut will affect the effectiveness of antibiotic treatment in mosquito microbiome research. Utilization of antibiotics may also perturb the community structure by only acting on sensitive bacteria. In summary, Table S4 presents the genes in the category of antibiotic resistance, efflux pumps and heavy metal detoxification, and the similarity to the orthologs in other taxa in the family Flavobacteriaceae.

The antibiotic resistance might have consequences for future work with the *E. anophelis.* Pathogenicity of the bacterium was recently demonstrated in a clinical case of meningitis in Africa [78] and a hospital outbreak in Singapore [79]. The isolates in both cases were resistant against a wide array of antibiotics. These case reports raised a concern regarding whether or not mosquitoes can pass *E. anophelis* and *E. meningoseptica* to humans in clinical situations and when handling mosquitoes in research. Further investigation is required to evaluate these potential risks.

### Mevalonate Pathway Utilization for Isoprenoid Synthesis

Isoprenoids comprise the largest group of organic molecules in nature and are involved in a wide variety of biological functions. The universal isoprenoid precursor and building block isopentenyl pyrophosphate (IPP) and its isomer dimethylallyl pyrophosphate (DMAPP) can be synthesized by two independent pathways, the classical mevalonate (MVA) pathway and the alternative 2C-methyl-D-erythtritol 4-phosphate (MEP) pathway. While the MVA pathway is present in higher eukaryotes, plant cytoplasm and archaea, the MEP pathway is generally found in eubacteria and the plastids of plants and apicomplexan parasites. Remnants of enzyme sequences for either pathway are commonly found among bacterial genomes, and in a few bacteria both pathways are active (reviewed in [80]).

**Figure 6:**
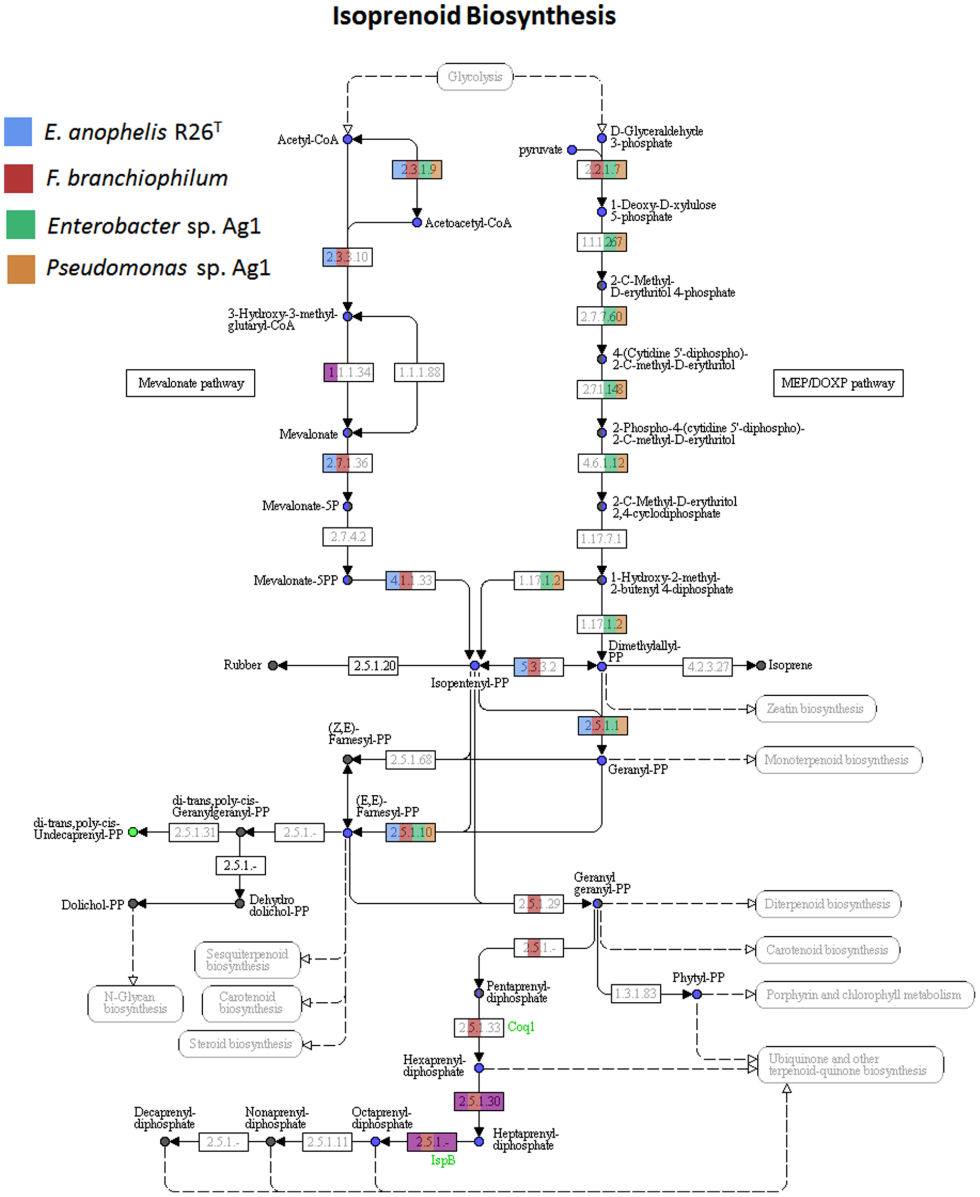
Isoprenoid synthesis pathway in four bacterial species. The color code represents the enzymes that are present in the species.

Interestingly, opposite to most eubacteria, *E. anophelis* and flavobacteria appear to utilize the MVA pathway for isoprenoid biosynthesis. Exemplified in Fig. 6, *E. anophelis* and *F. branchiophilum* possess all but one of the enzymes known in the MVA pathway and none in the MEP pathway, while *Enterobacter* sp. Ag1 and *Pseudomonas* sp. Ag1 have all enzymes in the MEP pathway. A comparison with six other related bacteria indicated this to be a general feature within the family Flavobacteriaceae (Table S6). Whereas the phosphomevalonate kinase (EC 2.7.4.2) seems to be missing, which is not uncommon in bacteria, the phosphorylation of phosphomevalonate is likely mediated via an alternate route in this family [81], [82]. A nonorthologous kinase in the galactokinase/homoserine kinase/mevalonate kinase/phosphomevalonate (GHMP) family could possibly play the role [82], [83]. In the *E. anophelis* genome, a homoserine kinase coding gene (D505_ 04189) and a galactokinase coding gene (D505_05014) were identified. Vinella *et al*. showed that the function of the MEP pathway is dependent on the ability to form Fe-S complexes in the IspG and IspH enzymes [84]. Fe-S complexes can be destroyed by nitric oxide (NO) [85], suggesting that possession of the MVA pathway would be conducive to living in a NO-rich environment. Because the production of NO is an essential part of both the mosquito anti-parasitic and anti-bacterial response [86]–[88], the MVA pathway utilizing *E. anophelis* and *E. meningoseptica* could have an advantage over other bacteria which have the MEP pathway in the mosquito midgut populations.

### Antioxidant Capacity

Hematophagous mosquitoes take a blood meal for egg production, the digestion of which changes the gut conditions drastically. Catabolism of hemoglobins results in the release of a large quantity of free heme in the gut lumen. The pro-oxidation of heme increases oxidative stress in the gut environment (reviewed in [89]). A gene encoding the heme-degrading protein HemS (D505_03742) was present in the genome with orthologs found in *E. meningoseptica* and *C. gleum*, but not in *F. branchiophilum*. The HemS protein has been shown to degrade heme *in vitro* and is required for the defense against oxidative stress upon hydrogen peroxide exposure in *Bartonella henselae* [90]. The genome also encompasses four hemolysins, one hemolysin D secretion protein and six hemolysin translocator HlyD proteins. These gene products may assemble hemolysin transporters, as demonstrated in *E. coli* [91], [92]. Hemolytic activity has been demonstrated in the fish pathogen *F. psychrophilus* [93] and *E. meningoseptica* [94]. In agreement with these findings, we found that *E. anophelis* also displays hemolytic activity and the ability to grow in the blood agar (Fig. S5). Following 24 h of incubation, *E. anophelis* caused a distinct brown discoloration of the blood agar (Fig. S5A). Despite a somewhat different appearance compared to *Streptococcus pneumonia* (Fig. S5B), the discoloration caused by either of these strains is a typical sign of α-hemolysis. Additional incubation for 24 h resulted in a clear, brown-colored zone adjacent to the growing *E. anophelis* bacteria. This effect was dependent on the bacterial density as single colonies caused discoloration but no clearance of the agar at this time point. Upon overall comparison, the peptide sequences of the orthologous genes were found with high similarity in *E. meningoseptica*, *C. gleum,* and to a less extent in the more divergent *F. branchiophilum*, indicating a broad conservation within the family Flavobacteriaceae. It is possible that the presence of hemolysins and heme degrading genes aids in the mosquito blood meal digestion. Similar synergism has been observed in *Ae. aegypti* [6].

Prokaryotic cells employ two redox-sensing regulons, OxyR and SoxRS, to sense oxidative stress signals and subsequently activate defense mechanisms [95]. The SoxRS regulon has not been found in *Bacteroidetes* [96], [97]. The *oxyR* gene (D505_12281) is, however, present in the *E. anophelis* genome and is located in proximity to the antioxidant genes *catalase* (D505_12286) and *manganese superoxide dismutase (MnSOD)* (D505_12271). This genomic arrangement is unique to *E. anophelis*; no synteny was found in other flavobacteria in this study, although *oxyR* was found in other flavobacteria. In addition, other genes in the oxidative stress category are present, including those encoding alkyl hydroperoxide reductases (AhpC/F), DNA-binding protein from starved cells (Dps), thioredoxins (Trxs), glutaredoxins (Grxs) and glutathione (GSH) peroxidase. OxyR controls the expression of *catalase, ahpC, ahpF* and *dps* in response to H_2_O_2_ [97], [98]. The genetic antioxidant capacity may contribute to the persistence of *E. anophelis* in the gut [2].

## Summary

The genome annotation provides insights into the capabilities of *E. anophelis*, and sheds light on its symbiotic relationships with the mosquito host and other members of the microbiome. The predominance of *E. anophelis* in the gut ecosystem of mosquitoes may represent an evolutionary fit. Firstly, the presence of a large number of TonB dependent receptors with numerous substrate specificities endows the bacteria with a sufficient capacity to acquire and utilize various biopolymers. Particularly, the polysaccharide utilization system supplies consumable carbohydrates for both microbial residents and host following the intake of nectar as well as cellulose. Secondly, the multidrug efflux pump genes of the RND and MATE superfamilies allow the bacteria to extrude heavy metals, microbicides and other toxic chemical compounds and maintain a homeostatic internal environment. Various antibiotic resistance mechanisms may serve as guardians for maintaining community structure in the gut microbial community. The interactions among antibiotic-producing and resistant bacteria may be one of the determinants that shape and stabilize the community structure. Similar metagenomic contexts have been demonstrated in natural environments [75] and host associated microbiomes [76], [77]. On the other hand, as an opportunistic pathogen, the multidrug resistance of *E. anophelis* renders infections very troublesome. Finally, the antioxidant capacity endows *E. anophelis* with defense against oxidative stress associated with blood digestion. The genome database provides a reference for further characterization of the mosquito gut microbiome and its impact on mosquito life traits.

## Supporting Information

Figure S1 **PCR verification of the putative phage insert in Ag1 not present in R26^T^.** (A) Primers were designed to flank the insertion sites of a phage like segment in Ag1. Primer pairs F1–R1 and F2–R2 were expected to yield 650 and 500 bp amplicons, respectively, in Ag1. The F1–R2 pair was expected to yield a 566 bp amplicon in R26, whereas no amplification was expected in Ag1 due to the large size of the insert (>35 kb). (B) Expected PCR products and sizes were confirmed using agarose gel electrophoresis.
(TIF)

Figure S2 **Phylogenetic relationship of** ***E. anophelis*** **relative to the taxa in the family** ***Flavobacteriaceae*** **inferred from 16 S ribosomal DNA (A) and rpoB gene (B).** Numbers above clades are bootstrap values (1000 replicates). The trees were constructed by Neighbor Joining criterion implemented in MEGA 5.1.
(TIF)

Figure S3 **Graphic view of conserved domains in TonB dependent transporters.** The protein ID (GenBank accession #) was given for each protein. Detailed domain information can be found in NCBI Conserved Domains database.
(TIF)

Figure S4 **Drug resistance and growth inhibitory capacity of** ***E. anophelis*** **.** Representative figure of the drug resistance displayed by *E. anophelis* R26^T^. Plates were cast using a mixture of 5×10^4^ bacteria in 6 ml Lysogeny broth and 1% SeaPlaque agarose (FMC BioProducts). Ciprofloxacin (100 µg/ml) and Cecropin A (100 µM) were added in a two-fold dilution series counter-clockwise in 2 mm wide holes with the lowest dose in the center and allowed to diffuse at ambient temperature for 30 min before incubation at 37°C for 24 h or until growth was apparent. *E. coli* strain D31 was used for comparison.
(TIF)

Figure S5 ***E. anophelis*** **displays α-hemolytic activity.** (A) Representative images taken from above (left plate) or underneath (right plate) at 48 h post inoculation. Black and white arrows depict the brown discoloration and clearance of the blood agar, respectively that were observed adjacent to the bacteria. The density dependence of the clearance zone was clear when observing the plate from underneath (right plate, white arrow). The right panel depicts the brown discoloration caused by individual colonies. (B) Control strains displaying the different types of hemolysis.
(TIF)

Table S1 **Draft genome statistics.**
(XLSX)

Table S2 **Coding sequence comparison between R26^T^ and Ag1 of** ***E. anophelis*** **.**
(XLSX)

Table S3 **Carbohydrate-active-enzyme proteins in the** ***E. anophelis*** **genome.**
(XLSX)

Table S4 **Drug resistance genes in** ***E. anophelis*** **and similarity to related genomes.** Numbers reflect percent identity of the best hit in related genomes to each gene in R26^T^. *E. m*., *Elizabethkingia meningoseptica*; *C. g.*, *Chryseobacterium gleum*; *F. b., Flavobacterium branchiophilum*.
(XLSX)

Table S5 **Sequence ID used in** **Figure 6** **.** Homologous sequences from related taxa and GenBank accession numbers were provided.
(XLSX)

Table S6 **Mevalonate pathway genes in different species.** *E. m*., *Elizabethkingia meningoseptica*; *C. g.*, *Chryseobacterium gleum*; *F. b., Flavobacterium branchiophilum*; *R. a.*, *Riemerella anatipestifer*; *F. j.*, *Flavobacterium johnsoniae* UW101; *C. sp.*, *Chryseobacterium* sp. CF314; *F. ba.*, *Flavobacteriaceae bacterium* 3519-10; *F. sp.*, *Flavobacterium* sp. F52.
(XLSX)
