# Correction: Insights from the Genome Annotation of Elizabethkingia anophelis from the Malaria Vector *Anopheles gambiae*

**DOI:** 10.1371/journal.pone.0122154

**Published:** 2015-03-18

**Authors:** The PLOS ONE Staff

There are errors in the Funding section. The correct funding information is as follows: JX was supported by grants (SC2GM092789, SC1AI112786) from the National Institute of General Medical Sciences and the National Institute of Allergy and Infectious Diseases of the National Institutes of Health, and the grant (DMS-1222592) from National Science Foundation. MS was an undergraduate research scholar supported by the NMSU Howard Hughes Medical Institute (HHMI) research scholars program. IF was supported by a grant from the Research Infrastructure—Integrating Activity project supported by the European Community (INFRAVEC) FP7 Infrastructures (grant agreement number: 228421). The funders had no role in study design, data collection and analysis, decision to publish, or preparation of the manuscript.
