# Supplementary figures and images for: Insights from the Genome Annotation of Elizabethkingia anophelis from the Malaria Vector Anopheles gambiae
Source: PLoS One. 2014 May 19;9(5):e97715. doi: 10.1371/journal.pone.0097715 (PMC4026382; doi:10.1371/journal.pone.0097715)

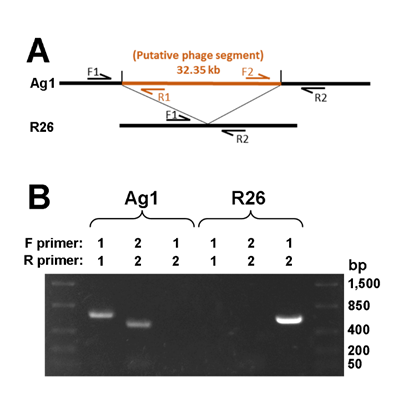

Supplement: Figure S1 — PCR verification of the putative phage insert in Ag1 not present in R26T. (A) Primers were designed to flank the insertion sites of a phage like segment in Ag1. Primer pairs F1–R1 and F2–R2 were expected to yield 650 and 500 bp amplicons, respectively, in Ag1. The F1–R2 pair was expected to yield a 566 bp amplicon in R26, whereas no amplification was expected in Ag1 due to the large size of the insert (>35 kb). (B) Expected PCR products and sizes were confirmed using agarose gel electrophoresis. (TIF) [file pone.0097715.s001.tif]

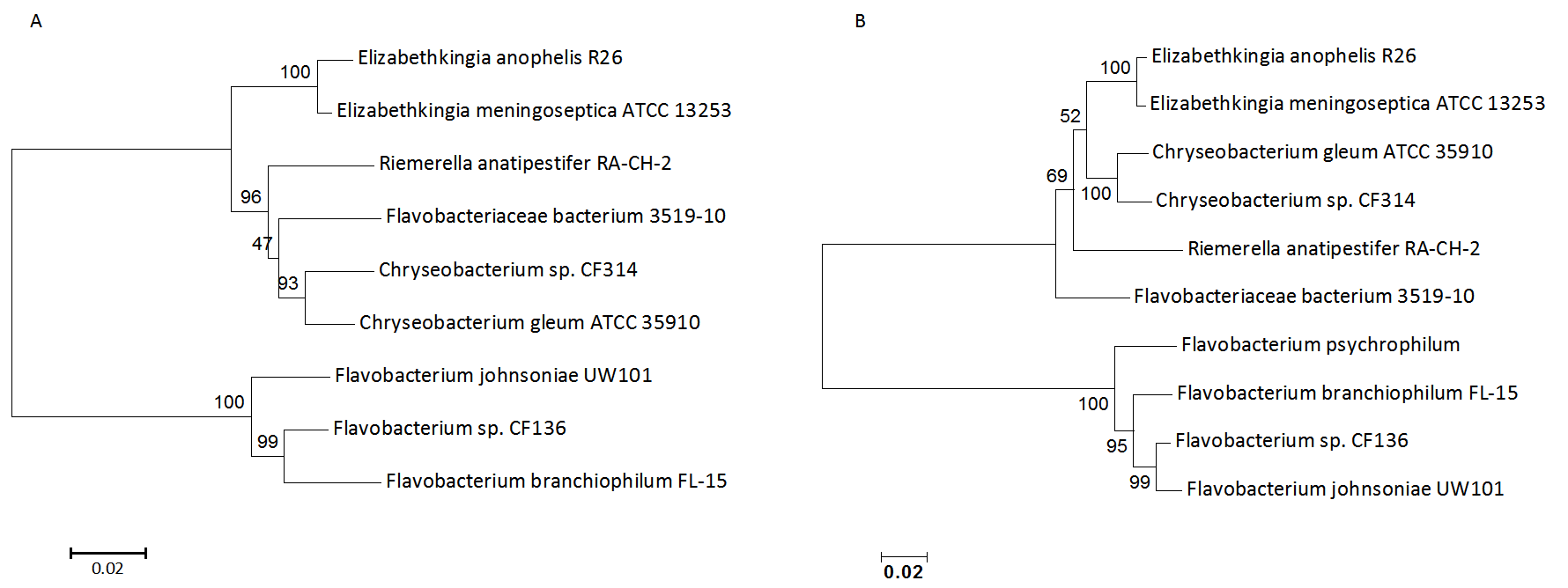

Supplement: Figure S2 — Phylogenetic relationship of E. anophelis relative to the taxa in the family Flavobacteriaceae inferred from 16 S ribosomal DNA (A) and rpoB gene (B). Numbers above clades are bootstrap values (1000 replicates). The trees were constructed by Neighbor Joining criterion implemented in MEGA 5.1. (TIF) [file pone.0097715.s002.tif]

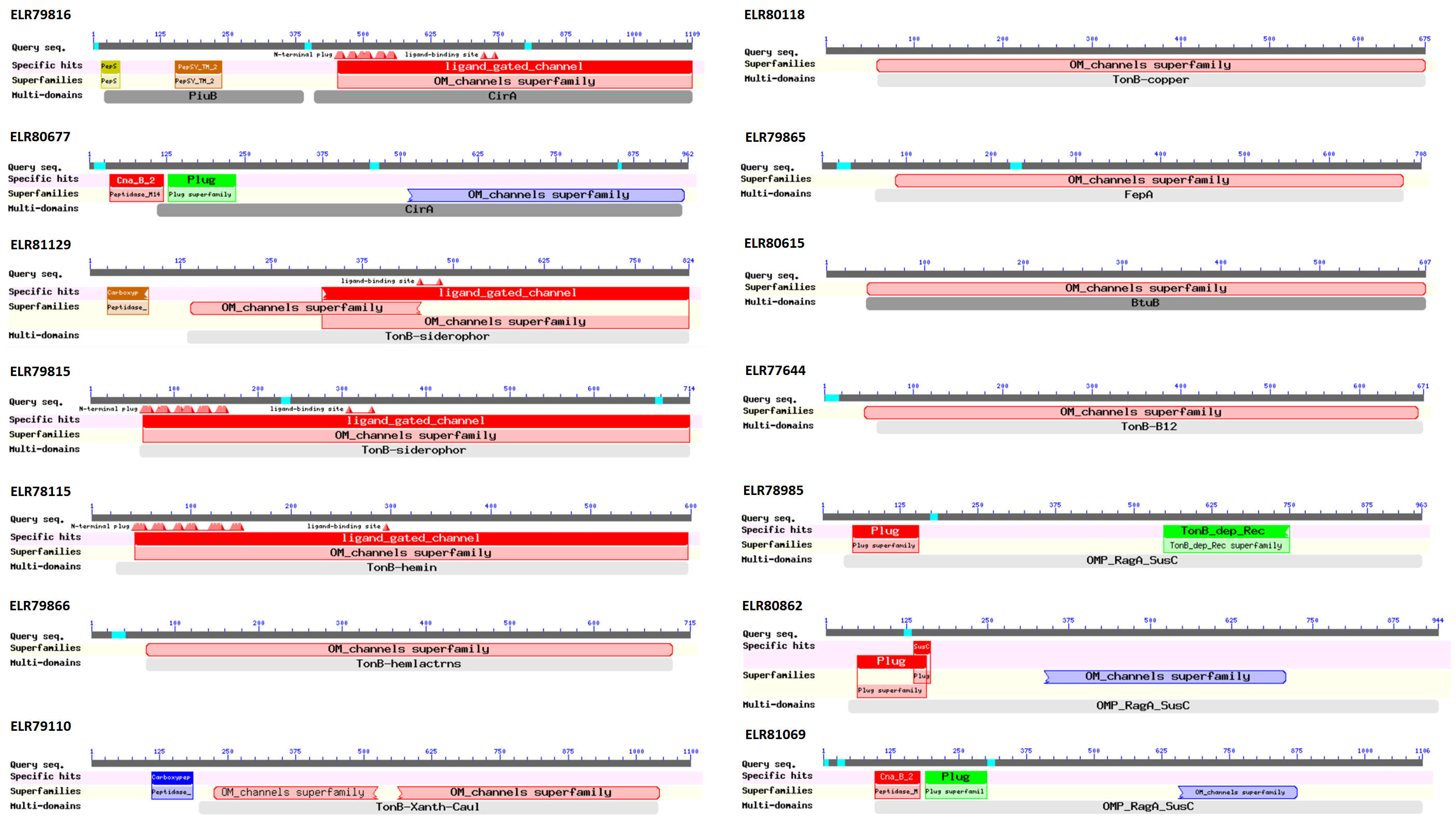

Supplement: Figure S3 — Graphic view of conserved domains in TonB dependent transporters. The protein ID (GenBank accession #) was given for each protein. Detailed domain information can be found in NCBI Conserved Domains database. (TIF) [file pone.0097715.s003.tif]

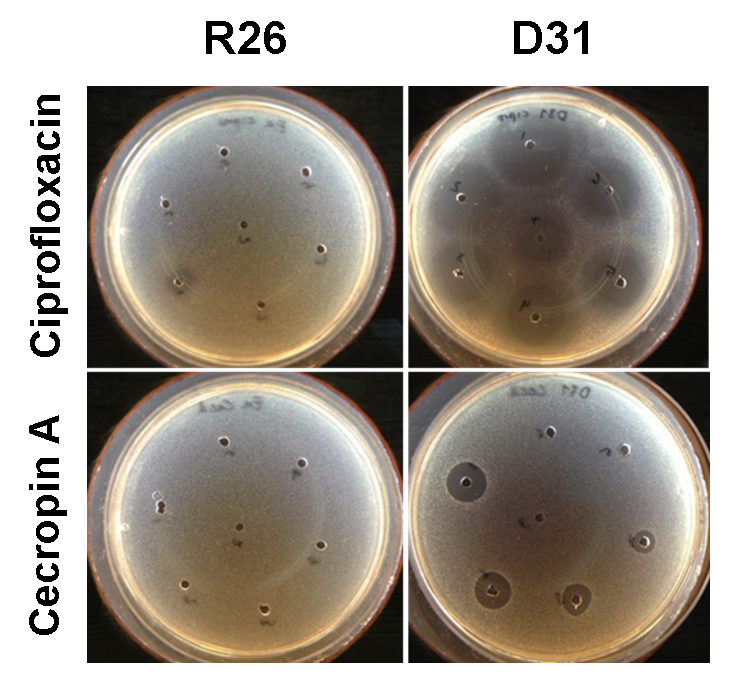

Supplement: Figure S4 — Drug resistance and growth inhibitory capacity of E. anophelis . Representative figure of the drug resistance displayed by E. anophelis R26T. Plates were cast using a mixture of 5×10^4^ bacteria in 6 ml Lysogeny broth and 1% SeaPlaque agarose (FMC BioProducts). Ciprofloxacin (100 µg/ml) and Cecropin A (100 µM) were added in a two-fold dilution series counter-clockwise in 2 mm wide holes with the lowest dose in the center and allowed to diffuse at ambient temperature for 30 min before incubation at 37°C for 24 h or until growth was apparent. E. coli strain D31 was used for comparison. (TIF) [file pone.0097715.s004.tif]

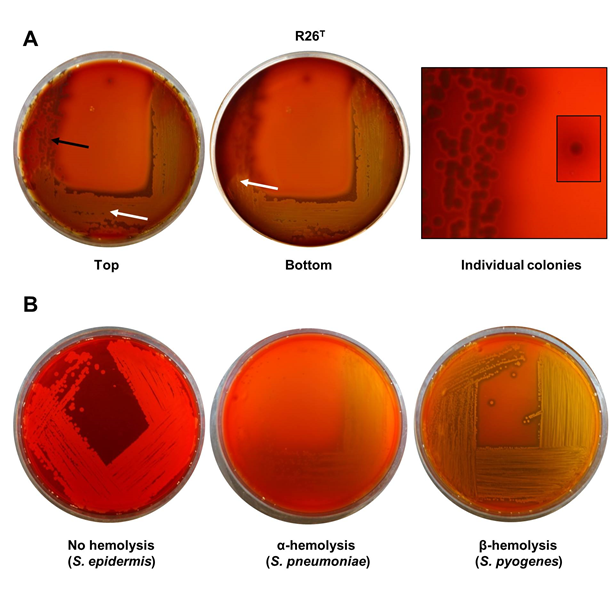

Supplement: Figure S5 — E. anophelis displays α-hemolytic activity. (A) Representative images taken from above (left plate) or underneath (right plate) at 48 h post inoculation. Black and white arrows depict the brown discoloration and clearance of the blood agar, respectively that were observed adjacent to the bacteria. The density dependence of the clearance zone was clear when observing the plate from underneath (right plate, white arrow). The right panel depicts the brown discoloration caused by individual colonies. (B) Control strains displaying the different types of hemolysis. (TIF) [file pone.0097715.s005.tif]
